# Heritability of circle of Willis variations in families with intracranial aneurysms

**DOI:** 10.1371/journal.pone.0191974

**Published:** 2018-01-29

**Authors:** Mayte Sánchez van Kammen, Charles J. Moomaw, Irene C. van der Schaaf, Robert D. Brown, Daniel Woo, Joseph P. Broderick, Jason S. Mackey, Gabriël J. E. Rinkel, John Huston, Ynte M. Ruigrok

**Affiliations:** 1 Department of Neurology and Neurosurgery, University Medical Centre Utrecht, Utrecht, the Netherlands; 2 Department of Neurology and Rehabilitation Medicine, University of Cincinnati, Cincinnati, Ohio, United States of America; 3 Department of Radiology, University Medical Centre Utrecht, Utrecht, the Netherlands; 4 Department of Neurology, Mayo Clinic, Rochester, Minnesota, United States of America; 5 Department of Neurology, Indiana University School of Medicine, Indianapolis, Indiana, United States of America; 6 Department of Radiology, Mayo Clinic, Rochester, Minnesota, United States of America; Universitatsklinikum Freiburg, GERMANY

## Abstract

**Background:**

Intracranial aneurysms more often occur in the same arterial territory within families. Several aneurysm locations are associated with specific circle of Willis variations. We investigated whether the same circle of Willis variations are more likely to occur in first-degree relatives than in unrelated individuals.

**Methods:**

We assessed four circle of Willis variations (classical, A1-asymmetry, incomplete posterior communicating artery and fetal circulation) in two independent groups of families with familial aneurysms and ≥2 first-degree relatives with circle of Willis imaging on MRA/CTA. In each (index) family we determined the proportion of first-degree relatives with the same circle of Willis variation as the proband and compared it to the proportion of first-degree relatives of a randomly selected unrelated (comparison) family who had the same circle of Willis variation as the index family’s proband. Concordance in index families and comparison families was compared with a conditional logistic events/trials model. The analysis was simulated 1001 times; we report the median concordances, odds ratios (ORs), and 95% confidence intervals (95%CI). The groups were analysed separately and together by meta-analysis.

**Results:**

We found a higher overall concordance in circle of Willis configuration in index families than in comparison families (meta-analysis, 244 families: OR 2.2, 95%CI 1.6–3.0) mostly attributable to a higher concordance in incomplete posterior communicating artery (meta-analysis: OR 2.8, 95%CI 1.8–4.3). No association was found for the other three circle of Willis variations.

**Conclusions:**

In two independent groups of families with familial aneurysms, the incomplete PcomA variation occurred more often within than between families suggesting heritability of this circle of Willis variation. Further studies should investigate genetic variants associated with circle of Willis formation.

## Introduction

Intracranial aneurysms are weak pouches in the walls of brain arteries that occur in 3.2% of the population [1]. Intracranial aneurysms are located at the circle of Willis, a circulatory anastomosis of arteries located at the base of the brain. Variation in the configuration of the circle of Willis is common. An incomplete circle of Willis, in which some of its arterial components are absent or hypoplastic, is seen in approximately 70% of individuals [2].

Rupture of intracranial aneurysms causes subarachnoid haemorrhage (aSAH), a type of stroke with a high case fatality and morbidity [3]. Individuals with first-degree relatives (FDRs) who have had an aSAH have an increased risk of aSAH and of developing unruptured intracranial aneurysms [4,5]. Aneurysms are more likely to occur in the same arterial territory in the circle of Willis within affected members of the same family than in affected individuals from unrelated families [6]. Aneurysm location may be influenced by variation in the configuration of the circle of Willis. Aneurysms of the anterior communicating artery (AcomA) have been associated with asymmetry of the proximal anterior cerebral arteries (A1 segments) [7–9], and with a lower frequency of fetal type posterior circulation [7], whereas aneurysms of the posterior communicating artery (PcomA) have been associated with a higher frequency of fetal type posterior circulation [7–9].

We hypothesized that the same variations in the configuration of the circle of Willis occur more often within families than between unrelated individuals. To test our hypothesis, we examined circle of Willis variations among two independent groups of families with a family history of intracranial aneurysms. We determined whether the circle of Willis configurations of FDRs of a designated member (proband) of a family (index family) were more likely to be the same as that proband’s circle of Willis configuration than the configurations of the FDRs of an unrelated family (comparison family).

## Methods

### Study populations

We analysed two independent groups of families with familial aneurysms. The first group (group 1) included families screened at the University Medical Centre Utrecht in the Netherlands. The second group (group 2) included North American, New Zealander, and Australian families from the Familial Intracranial Aneurysm (FIA) Study [10]. A general waiver for informed consent was obtained from the Institutional Review Board of the University Medical Centre Utrecht and of the Mayo Clinic, MN.

#### Group 1

At the University Medical Centre Utrecht, data on individuals with a family history of aSAH are prospectively recorded in a database. Screening for intracranial aneurysms is offered to individuals with two or more FDRs with a history of aSAH or unruptured intracranial aneurysm [11]. The standard imaging modality is magnetic resonance angiography (MRA). In case of claustrophobia or previously clipped aneurysm, computed tomography angiography (CTA) is performed instead. For the current analysis, we used all available information from April 1993 to February 2015.

#### Group 2

In the FIA study, families with familial aneurysms from 26 clinical centres in North America, New Zealand, and Australia were recruited partly prospectively and partly retrospectively between 2002 and 2012, according to previously described inclusion criteria [10]. Imaging of family members was performed prospectively. Screening for aneurysms by MRA was offered to individuals with an affected FDR (with a history of aSAH or unruptured intracranial aneurysm) and any of the following risk factors: age 30 years or over, history of hypertension, mean blood pressure of >140 mmHg systolic or >90 mmHg diastolic, and/or a 10 pack-year history of smoking. We used all available information from the FIA study. All available imaging consisted of MRAs.

### Data collection

**Fig 1:**
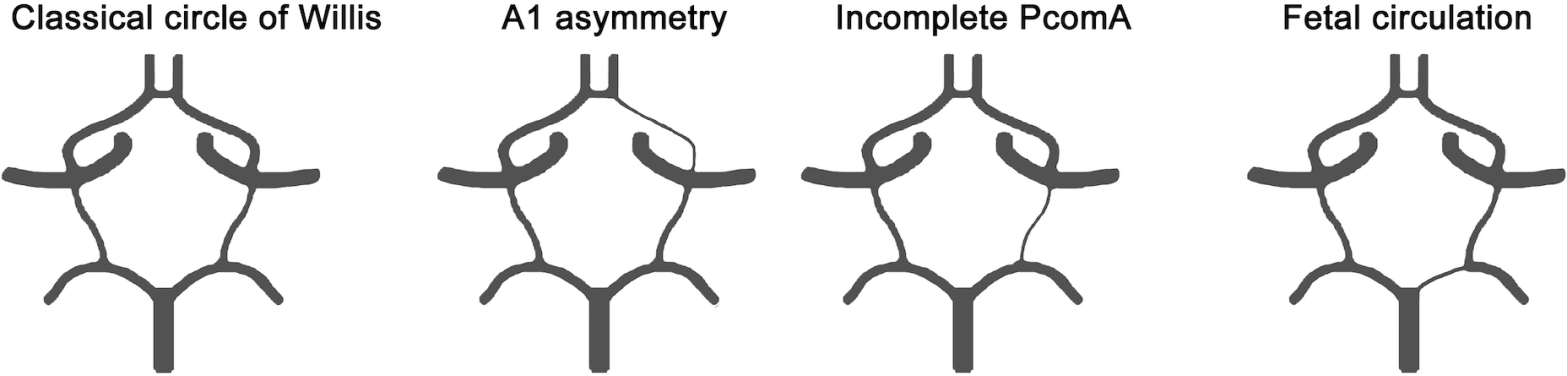
The four categories of circle of Willis variations used in this study. Classical circle of Willis was defined as A1, P1 and PcomA diameters >0.8 mm, with no asymmetric A1s and no fetal posterior circulation. A1 asymmetry was defined as a difference in diameter of the A1 segments of >33%. Incomplete PcomA was defined as a uni- or bilateral PcomA diameter <0.8 mm. Finally, fetal posterior circulation was defined as a PcomA diameter >10% larger than the P1 diameter on the same side. A1 = proximal segment of anterior cerebral artery; P1 = proximal segment of posterior cerebral artery; PcomA = posterior communicating artery.

The present analysis included all families in which at least two FDRs had imaging of the circle of Willis by MRA or CTA, irrespective of whether or not individuals had a history of aSAH or unruptured aneurysms. Within each family, the proband was defined as the family member with the highest number of imaged FDRs who was first brought under medical attention (group 1) or first recruited by the FIA study (group 2). Thus, the proband was not necessarily carrier of an aneurysm. Subjects who had a CTA with >1mm slice thickness or digital subtraction angiography only were excluded. Subjects with poor quality imaging, for example due to scattering from an aneurysm clip or due to movement, were excluded as well. For the families of group 1, scans were analysed in Philips Intellispace Portal; scans from group 2 were analysed in GE Advantage Windows. Subjects’ most recent scans were analysed in MIP reconstruction. After obtaining a three-dimensional view of the vessel, the average diameter of the following arteries of the circle of Willis was measured proximally on the vessel segment: both A1 segments, both proximal posterior cerebral arteries (P1 segments), and both PcomAs. All MRAs and CTAs were blindly reviewed by the same observer (MSK), who was supervised by two experienced neuroradiologists (ICS for group 1, JH for group 2). Circle of Willis variations were grouped into four categories as previously described [12] and shown in Fig 1: classical circle of Willis, A1 asymmetry, fetal posterior circulation and incomplete PcomA.

### Statistical analysis

We used a conditional logistic events/trials model used in a previous analysis that examined the locations of intracranial aneurysms among affected individuals within the same family [6]. Within each family unit, we calculated the proportion of FDRs who had the same circle of Willis variation as the designated proband of the family unit (“total” concordance proportion). In addition, we calculated concordance proportions separately for the four circle of Willis variations. Each family unit was then randomly matched to an unrelated family, selected from the group of families whose proband’s circle of Willis variation was different from that of the proband of the index family. Selection was done with replacement, i.e. each family could be selected more than one time. The proportion of FDRs of the comparison family who had the same circle of Willis variation as the proband of the index family was calculated. Table 1 shows a hypothetical example of the determination of concordance proportions between the index proband and FDRs of the index family and between the index proband and FDRs of the comparison family. The concordance proportions in the index and comparison families were compared using a conditional logistic events/trials regression model. This process was performed 1001 times to obtain a more robust analysis, resulting in 1001 sets of concordance proportions, odds ratios (ORs) and 95% confidence intervals (95% CIs). Results are the medians of these 1001 calculations.

**Table 1:** Sample calculation of concordance proportions for index and comparison family units.

**Index family unit: proband and FDRs**	**Circle of Willis variation(s)**	**Same circle of Willis variation as index proband?**		
		**Incomplete PcomA**	**A1** **asymmetry**	**Total**
Proband (female)	Incomplete PcomA, A1 asymmetry			
Brother	Classical circle	no	no	no
Daughter 1	Incomplete PcomA	yes	no	yes
Son 1	Incomplete PcomA, A1 asymmetry	yes	yes	yes
Son 2	Fetal PC	no	no	no
Daughter 2	Incomplete PcomA	yes	no	yes
**Concordance proportion**		**3/5**	**1/5**	**3/5**
**Comparison family unit: proband and FDRs**	**Circle of Willis variation(s)**	**Same circle of Willis variation as index proband?**		
		**Incomplete PcomA**	**A1** **asymmetry**	**Total**
Proband (male)	Classical circle			
Brother 1	A1 asymmetry, fetal PC	no	yes	yes
Brother 2	Classical circle	no	no	no
Brother 3	Incomplete PcomA	yes	no	yes
Sister	Classical circle	no	no	no
**Concordance proportion**		**1/4**	**1/4**	**2/4**

Family units shown here are hypothetical. Note that the index proband is not compared with the comparison proband because the circle of Willis configuration of the comparison proband is different, by definition, from that of the index proband. Thus, inclusion of the comparison proband would result in biased (lower) concordance proportions—in this example, 1/5, 1/5, and 2/5 rather than 1/4, 1/4, and 2/4.

A1 = proximal segment of anterior cerebral artery; FDR = first-degree relative; fetal PC = fetal posterior circulation; PcomA = posterior communicating artery.

We first analysed both groups separately and then performed an inverse variance fixed effects meta-analysis if data allowed to do so. We assessed for heterogeneity using the Higgins *I*^2^ [13]. Mild to moderate heterogeneity was defined as *I*^2^≤60%, substantial heterogeneity as *I*^2^>60%.

## Results

### Group 1

We found 122 families with at least two FDRs who had imaging of the circle of Willis. Twelve FDRs from 10 different families were excluded because of poor imaging quality. Of the 122 probands, 76 had one FDR with imaging of the circle of Willis, 25 had two, six had three, four had four, four had five, three had six, two had seven, one had 11, and one had 14 FDRs with imaging of the circle of Willis.

The prevalence of each circle of Willis variation among probands and FDRs is given in Table 2. Of the 122 probands, 78 (63.9%) were women; 27 (22.1%) had a history of aSAH, 17 (13.9%) had unruptured aneurysms, and no aneurysms were identified upon screening in the remaining 78. Of the 258 FDRs, 112 (43.4%) were women; 11 (4.3%) had a history of aSAH, and 27 (10.5%) had unruptured aneurysms.

**Table 2:** Prevalence of circle of Willis variations in probands and FDRs.

Circle of Willis variation	Group 1		Group 2	
	122 probands n (%)	258 FDRs n (%)	122 probands n (%)	188 FDRs n (%)
Classical circle	25 (20.5)	47 (18.2)	26 (21.3)	28 (14.9)
A1 asymmetry	21 (17.2)	67 (26.0)	21 (17.2)	44 (23.4)
Incomplete PcomA^a^	75 (61.5)	156 (60.5)	78 (63.9)	124 (66.0)
Fetal PC^a^	29 (23.8)	73 (28.3)	29 (23.8)	44 (23.4)

Individuals can have more than one circle of Willis variation. Prevalences did not differ significantly between probands and FDRs of any group for all circle of Willis variations (chi square; p = 0.543).

^a^ Same variation bilaterally is counted only once.

A1 = proximal segment of anterior cerebral artery; FDR = first-degree relative; fetal PC = fetal posterior circulation; PcomA = posterior communicating artery.

Overall, the concordance in circle of Willis variations was higher for the index families than for the comparison families (Table 3). Of the four different circle of Willis variations, only incomplete PcomA occurred statistically significantly more often within the same family.

**Table 3:** Concordance proportions in index and comparison families and odds ratios for group 1, group 2 and meta-analysis.

Circle of Willis variation	Group 1			Group 2			Meta-analysis^b^	
	Index families	Comparison families^a^	OR (95% CI)^a^	Index families	Comparison families^a^	OR (95% CI)^a^	OR (95% CI)	Heterogeneity (I^2, %)
Classical circle	33.8%	19.7%	2.2 (0.8–6.0)	24.7%	21.0%	1.2 (0.4–3.3)	1.7 (0.8–3.4)	0
A1 asymmetry	25.1%	21.9%	1.1 (0.4–3.4)	20.3%	20.1%	1.1 (0.3–4.0)	1.1 (0.5–2.5)	0
Incomplete PcomA	65.5%	43.2%	2.9 (1.7–5.2)	64.3%	39.2%	2.7 (1.4–5.1)	2.8 (1.8–4.3)	0
Fetal PC	36.1%	20.7%	2.3 (0.9–5.9)	25.3%	25%	1.0 (0.4–2.9)	1.5 (0.8–3.1)	28
Any variation	52.3%	35.3%	2.5 (1.6–3.9)	48.6%	35.5%	1.8 (1.1–2.9)	2.2 (1.6–3.0)	0

^a^ Values represent medians of 1001 iterations.

^b^ Inverse variance fixed effects model.

A1 = proximal segment of anterior cerebral artery; fetal PC = fetal posterior circulation; PcomA = posterior communicating artery.

### Group 2

In group 2, 123 families with at least two FDRs with imaging of the circle of Willis were identified. One family was excluded because of poor imaging quality in both imaged FDRs. One FDR of an included family was excluded because of poor imaging quality. Of the 122 remaining probands, 87 had one FDR, 20 had two FDRs, four had three, seven had four, three had five, and one had six FDRs with imaging of the circle of Willis; 76 (62.3%) were women; one (0.8%) had a history of aSAH, and 20 (16.4%) had unruptured aneurysms. Of the 188 FDRs, 103 (54.8%) were women, one (0.5%) had a history of aSAH, and 13 (6.9%) had unruptured aneurysms. Table 2 also shows the prevalence of each circle of Willis variation in group 2. Similarly to group 1, the concordance in circle of Willis variations was higher in index families than in comparison families, and of the separate variations only incomplete PcomA had a statistically significant higher occurrence within families (Table 3).

### Comparison and meta-analysis of groups 1 and 2

Heterogeneity between the two study groups was small with I^2^ values ranging from 0% to 28%. On combining the results of both groups by means of a meta-analysis we found a higher occurrence within families of circle of Willis variations overall and of the incomplete PcomA variation (Table 3). The classical circle and fetal posterior circulation (fetal PC) variations had mildly elevated ORs, but with wide confidence intervals and no statistical significance.

## Discussion

This study showed that the incomplete PcomA variation is more likely to occur within families than between families. No familial association was found for the classical variation, A1 asymmetry or fetal posterior circulation.

The formation of vascular anatomy is a highly complex process in which the embryonic vascular template, blood flow and signalling from surrounding tissues all play a role [14]. Little is known about the heritability of these processes. We did not find previous studies on the heritability of circle of Willis variations in humans. In gerbils, the same circle of Willis variations are seen in related gerbils [15], and genes associated with different circle of Willis configurations have been identified in these rodents [16]. In mice, knock-out of Notch signalling pathways led to lack of anterior anastomosis and a generalised loss of arterial symmetry in the circle of Willis, suggesting that these pathways may play a role in the occurrence of circle of Willis variations [17]. Future studies may analyse genetic risk factors associated with circle of Willis formation in humans. For example, in genome wide association study (GWAS) data of patients with intracranial aneurysms [18,19], patients with the incomplete PcomA variation may be compared to patients with other CoW variations.

Previous data suggest that certain anatomic variations in the circle of Willis predispose to the development of aneurysms at several locations [7–9]. Hypoplasia or absence of segments of the circle of Willis alter blood flow patterns [20], which may contribute to aneurysm formation. Higher hemodynamic shear stress and strong flow acceleration promote the development of aneurysms [21–23]. Among patients screened for familial aneurysms, those who developed aneurysms had significantly more hypoplastic adjacent circle of Willis segments on previous CTA or MRA than those who did not develop aneurysms [24]. AcomA aneurysms recurred more often after coiling in patients with unilateral A1 aplasia and basilar artery tip aneurysms recurred more often in patients with asymmetric basilar artery fusion [8].

For A1 asymmetry, the present study revealed low within-family concordance proportions (20%–25%), which were not significantly different from the between-family proportions (20%–22%). This circle of Willis variation is most often associated with aneurysms in the anterior cerebral artery (ACA) territory [7–9]. A previous study also showed low and similar concordance proportions between index and comparison families for location of aneurysms in the ACA territory (both 27%) [6]. The two studies confirm that the A1 asymmetry/ACA aneurysm association is unlikely to have a heritable component. We do not know if the high familial concordance in incomplete PcomA may partly explain the high familial concordance of aneurysms in certain other locations [6].

A strength of our study is that we validated our findings by studying two independent groups. The results are further confirmed by an almost absent heterogeneity between the two groups. Nevertheless, this study has several limitations. First, our sample consisted of subjects with familial aneurysms and it is unclear how these findings can be extrapolated to the general population. Second, different criteria for aneurysm screening were used in both groups. In group 1 screening was offered only to those with two or more affected FDRs, while in group 2 it was offered to anyone with one affected FDR and any additional risk factor such as older age, hypertension or smoking. In group 1 screening for additional aneurysms was performed also in patients with a known history of aSAH while in group 2 these patients were not routinely imaged. These different screening criteria between group 1 and 2 may explain the higher prevalence of history of aSAH in group 1 (10% versus 0.6% in group 2) and of intracranial aneurysms overall in group 1 (21.6% versus 11.3% in group 2). However, we do not feel this difference is likely to have influenced our results, also because the data on circle of Willis variations in the two groups are largely homogeneous. We do not think the partly retrospective recruitment of families in group 2 is likely to have influenced our results either, since imaging of the family members was performed prospectively. Third, we could not include sex as a covariate in our analysis. Sex is a potentially confounding factor, since women have been found to have a higher prevalence of fetal posterior circulation [9]. However, we suspect this did not affect our results because we did not find a familial occurrence of fetal posterior circulation; in addition, the proportion of women and men with this variation was comparable in our families. Fourth, it was not possible to perform the current analysis in our families for additional types of circle of Willis variations such as incomplete A1 or incomplete P1 because they appeared too rare. We have, however, included the most prevalent circle of Willis variations, including those variations associated with aneurysms. Fifth, other morphological aspects such as vascular angles may not have a heritable component [25]. This was not analysed in our study but may be subject for further future research. Sixth, the majority of the imaging studies used in this study were MRAs, although CTA has better spatial resolution than MRA. Last, our sample size was relatively small, which may have contributed to the wide confidence intervals for classical circle and fetal posterior circulation.

Our findings suggest that the incomplete PcomA variation has a heritable predisposition, which calls for future studies on genetic variants associated with circle of Willis formation. We do not know if the high familial concordance in incomplete PcomA may partly explain the high familial concordance of aneurysms in certain locations [4]; this should be the subject of further studies.

## Supporting information

S1 File Group 1 data. (XLSX)

S2 File Group 2 data. (XLSX)
